# COVID Vaccine Rollout for Older Adults in South Korea

**DOI:** 10.1093/geroni/igab046.713

**Published:** 2021-12-17

**Authors:** Chan Mi Park

**Affiliations:** Asan Medical Center, Songpa-gu, Seoul-t'ukpyolsi, Republic of Korea

## Abstract

It has just started in South Korea. We will provide detailed information later. The symposium has experts from 8 countries. We will use an interview and dialog format, instead of presentations.

